# Bullying as an Adverse Childhood Experience?—State of the Art

**DOI:** 10.3390/children13050609

**Published:** 2026-04-28

**Authors:** Lea Berger, Eva Möhler

**Affiliations:** Department of Child and Adolescent Psychiatry, Universität des Saarlandes, 66123 Saarbrücken, Germany

**Keywords:** ACE, bullying, children, trauma

## Abstract

**Highlights:**

**What are the main findings?**

- A compressive body of literature suggests that there are many similarities between ACEs and bullying regarding health outcomes and embodiment.
- Further evidence that bullying should be considered an ACE was found in studies on expanded ACEs, LCA and associations between bullying and ACEs.

**What is the implication of the main finding?**

- Bullying should be included in ACE measurements.
- More research is needed to replicate results and shed light onto the discrepancies between ACEs and bullying as well as in the underlying mechanisms of embodiment.

**Abstract:**

The objective of this state-of-the-art review was to ascertain whether bullying should be regarded as an ACE. To that end, a systematic search of the extant literature was conducted. A comprehensive search of three databases yielded 27 studies that met the inclusion criteria. The papers that were identified were sorted into the following categories: expanded ACE, associations between ACEs and bullying, latent class analysis, biological stress markers and negative health outcomes associated with ACEs and bullying. The extant evidence indicates that bullying falls within the ambit of the ACE concept. Moreover, bullying contributes independently and as part of the cumulative score to the prediction of negative health outcomes. However, minor discrepancies to other ACEs were identified, for example in the different path of embodiment regarding changes in BMI. It is recommended that subsequent studies incorporate bullying as an element of their ACE measurements. One approach to doing so would be to utilize the ACE-IQ. Further research is necessary to elucidate the embodiment of the consequences of ACEs and bullying.

## 1. Introduction

### 1.1. ACE

Adverse childhood experiences (ACEs) are defined as traumatic events that occur before the age of 18 [1,2,3]. These experiences have been shown to have a lasting impact on mental and physical health, as well as development [1,2,3]. The first study of ACEs was conducted by Felitti et al. at the CDC Kaiser Permanente study over two decades ago [3]. The 10 ACEs can be categorized into three distinct groups, abuse, household dysfunction and neglect, and comprise the following experiences: physical abuse, sexual abuse, emotional abuse, physical neglect, emotional neglect, witnessing household violence, substance abuse by a household member, separation or divorce of parents, mental illness of a household member, and incarceration of a household member. Exposure to ACEs has been demonstrated to result in numerous negative mental and physical health outcomes. ACEs were associated with cardiovascular diseases, cancer, and respiratory diseases [3]. It is evident that exposure to ACEs is a prevalent occurrence. A substantial meta-analysis undertaken by Hughes et al. established that 57% of individuals have encountered at least one ACE, with 13% having experienced four or more [4]. A recent study conducted in Germany revealed that the prevalence of slightly lower but comparable figures was observed, with 46% of cases exhibiting one or more ACE and 9% of cases demonstrating four or more ACEs [5]. As Dube demonstrates in their study, there is a robust correlation between exposure to ACEs and a range of problematic outcomes, including aggressive and antisocial behavior, substance misuse and sexual risk behavior [1]. Evidence of a dose-dependent relationship between the number of ACEs and negative outcomes was found [6]. The ACE model posits that exposure to stressful life events engenders psychological distress, which, in turn, precipitates adverse outcomes by inducing maladaptive changes in various emotional, cognitive, and physiological functions [7]. Furthermore, research indicates that exposure to one ACE is associated with an increased risk of exposure to another ACE [8].

### 1.2. Bullying

Bullying among children and adolescents is a pervasive issue that should be regarded as a public health concern [9,10]. The term “bullying” is defined as aggressive behavior directed towards a victim, intended to cause harm, and perpetuated over an extended duration while maintaining an imbalance of power between the perpetrator and the victim [11]. Such an imbalance of power can manifest in various forms. For example, the bully may exhibit superior physical strength relative to their victim. Alternatively, the bully’s social status may be evidenced by their greater social network or higher status within their peer group. Becoming a victim of bullying is associated with having a poor physical condition, lower mental well-being [12], the absence or low quality of friendships, less peer support [13] or contact to a deviant peer group [14], and coming from a low socioeconomic status family or a single parent household or a sexual or ethnic minority [15,16,17,18]. Bully perpetration has been demonstrated to be associated with engagement in illegal activities, delinquency or substance abuse [19,20] and exposure to violence in the family [16]. Bullies tend to be a little older compared to victims [21]. A number of factors have been identified as contributing to bullying involvement for both victim and bully roles. These include poor parenting and inadequate supervision, a problematic peer group or school environment [16], dissatisfaction with body weight and increased sedentary habits [21] and male gender [12,21,22]. Bullying can be categorized as either direct or indirect and can be further subdivided into the following forms: verbal bullying, threats, physical bullying, relational bullying, and social bullying [23]. The phenomenon of bullying can occur in both online and offline spaces, with the former being referred to as cyberbullying and the latter as traditional bullying. A comparison of cyberbullying and traditional face-to-face bullying reveals several distinguishing characteristics. Cyberbullying allows for anonymous bullying and it is harder to escape the bullying since it is not restricted to school [24]. The enlarged online audience can result in a proliferation of complex bystander behavior. These behaviors may take various forms, including inaction, the distribution of harmful content to others, active bullying of the victim, or the forwarding of content to the victim with the intention of informing and supporting them [25]. Moreover, cyberbullies appear to experience less remorse for their actions in comparison to their offline counterparts [25]. Due to these differences, it is under debate whether cyberbullying fulfills the criteria for bullying. The challenges to the definition primarily pertain to the criteria of repetition and an imbalance of power. An imbalance of power is usually defined as the bully having superior strength or popularity, but neither is necessary for cyberbullying. Repetition and further distribution of harmful content is a common feature of cyberbullying, but the phenomenon often escalates rapidly beyond the control of the bully. The applicability of the definition remains ambiguous in circumstances where repetition has not been initiated by the initial bully [26]. Nevertheless, a considerable degree of overlap exists between cyberbullying and offline bullying involvement [27]. The impact of bullying on children can be significant, with potential consequences for both their mental and physical well-being that may persist into adulthood [28]. A substantial body of research indicates a high correlation between victimization and subsequent mental disorders. Associations to bullying have been identified in the context of depression [29,30], anxiety [31], social phobia [32], suicidality and self-harm [33,34], body dysmorphic disorders, for example muscle dysmorphia [35,36], and psychosomatic problems, including headaches, insomnia, and stomach aches [37,38]. A further issue that has emerged as a consequence of bullying is school absenteeism and diminished academic performance [31,39]. A higher risk for being overweight and a higher body mass index (BMI) is observed in children who have been subjected to bullying [40]. It was hypothesized that bullying interrupts the development of healthy emotion regulation due to toxic stress and repeated negative emotions [41]. As indicated by various research, health problems resulting from bullying could be a consequence of changes in the neuroendocrine system and inflammatory processes [42,43].

Despite the serious consequences of bullying, the prevalence is high. According to the World Health Organization (WHO), 2–33% of children are subjected to peer victimization and on average 6% of adolescents reported that they bullied others [44]. A meta-analysis encompassing studies from Asia, Africa, Australia, Europe and North and South America revealed a prevalence of 25% for bullying victimization, 16% for bullying perpetration and 16% for bully victims [45]. A recent study conducted in Germany revealed that 14% of the population engaged in bullying as bully, victim or bully–victim, while 8.6% of individuals experienced victimization [46]. The occurrence of cyberbullying has been demonstrated to be less frequent, with a prevalence of 7%. Another study examined the prevalence of various forms of bullying [47]. The findings indicated that the most prevalent forms of bullying were those involving insults and name-calling due to appearance and weight (52%), followed by being ignored and excluded (45%), and physical bullying (8.6%). In total, 6.9% of the participants indicated that they had been subjected to bullying due to their race, constituting the least prevalent form of bullying [47].

### 1.3. Aim of the Review

There is a growing body of research recognizing bullying as an ACE. However, the definition of ACEs is not always consistent, and the term “ACE” is often used for a wide range of slightly different experiences. Additionally, consequences of ACEs are varied. The present state-of-the-art review aims to examine the extant evidence on the conceptualization of bullying as an ACE. Specifically, it seeks to address the question of whether bullying fits within the conceptual framework introduced by Felitti et al. [3] in terms of long-lasting health consequences that start in childhood and can persist into adulthood [48,49]. ACEs were linked to negative mental (e.g., depression, anxiety, hallucinations, panic reactions, sleep disorders, memory disturbance and poor anger control) [3,50,51,52,53] and physical (e.g., ischemic heart disease, cancer, chronic lung disease, skeletal fractures, liver disease, diabetes, respiratory disease) health outcomes [4,53,54,55,56]. They were found to be risk factors for behavioral health outcomes (e.g., smoking, substance abuse, obesity) [4,53,54,57]. Moreover, ACEs were associated with premature mortality [58]. These health outcomes often persist into adulthood. In order to respond to the question of whether bullying is an ACE, a comparison must be made to determine if analogous outcomes are observed for bullying. Additionally, it is necessary to ascertain whether the inclusion of bullying as an expanded ACE enhances the explanatory power of models that link childhood adversity to negative health outcomes. To further understand the underlying mechanisms between ACEs, bullying and health outcomes, paths of biological embodiment should be compared. Biological embodiment describes the process by which exposure to different psychosocial or environmental factors leads to measurable changes in physiological systems. For ACEs, changes in neuronal, endocrine, immune and metabolic physiology were observed [59].

## 2. Systematic Literature Search

**Figure 1:**
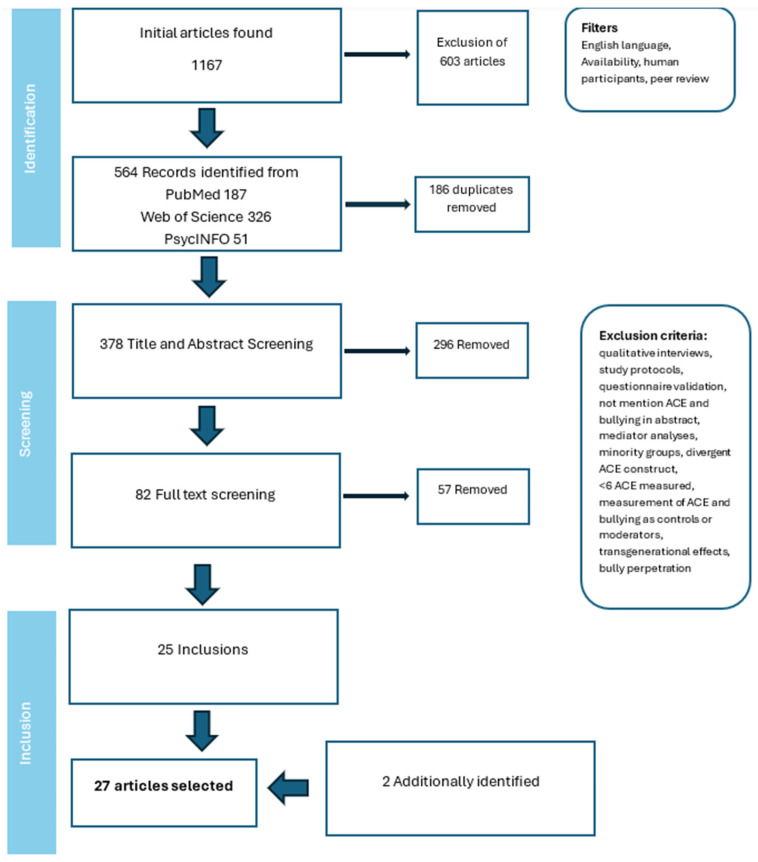
Overview of the screening and selection process.

The state-of-the-art review was conducted following the methodology suggested by Barry et al. [60]. A comprehensive search of the relevant literature was conducted in three data banks: PubMed (29 October 2025), PsycINFO and Web of Science (both 30 October 2025) using the following search term: “bullying AND (ACE OR adverse childhood experience) AND (children OR Child OR childhood OR youths OR adolescent)”. The terms “victimization”, along with “bullying”, was excluded because it yielded a substantial number of irrelevant results due to other forms of victimization. A total of 1167 papers were identified and filtered based on the following criteria: English language, availability, human participants, and peer review. It should be noted that there was no peer review filter available for Web of Science. Ultimately, 564 studies remained. Following the removal of the duplicates, a total of 378 papers were retained for the subsequent title and abstract screening. Following this initial screening, 82 studies were deemed to meet the inclusion criteria. The exclusion criteria encompassed the following: qualitative interviews, study protocols, questionnaire validation, mediator analyses of other variables, an ACE construct that was not aligned with the original ACE study, measurement of an incomplete number of ACEs (<6) and bullying and ACEs not mentioned in the abstract, studies that focused on bully perpetration, transmission effect studies, minority groups such as autistic or schizophrenic participants who significantly differ from non-pathological humans in their functioning, thinking, and perception, and studies that merely measured ACEs and bullying as part of their control variables. After full-text screening, 25 studies were included in this state-of-the-art review. Additionally, two studies were identified through hand searching. The initial screening and selection of studies were conducted by the first author and subsequently approved by the second author. Figure 1 provides an overview over the screening process and study selection. The quality rating of the included studies can be found in Table 1. For the quality rating, the mixed methods appraisal tool was employed [61]. For the study quality rating, the items for the category “Quantitative non-randomized studies” were selected according to the methods employed in the selected studies. Overall, most studies demonstrated commendable quality. However, some studies relied on non-representative samples. Another issue for some studies was missing outcome data, which may have introduced bias, but is not uncommon in psychological research.

Following comprehensive literature review, studies were consolidated into the following categories: extended ACE, associations between ACEs and bullying, latent class analysis, biological stress markers, and negative health outcomes associated with ACEs and bullying. The analysis was conducted in two steps, following stage 5 of the methodology suggested for state-of-the-art reviews [60]. Selected studies were reviewed in the first step. In the second step, historical context for the development of our understanding of ACEs and bullying was provided and the conclusions drawn in step one were contrasted with findings of other studies in the field to provide a compressive review of the state-of-the-art understanding regarding whether bullying should be included into the ACE framework.

## 3. Results

### 3.1. Literature Review

#### 3.1.1. Expanded ACE

A total of eight studies were identified in the “Expanded ACE” category. One study was a review, two employed factor analysis, and four studies used a regression method to study the impact of expanded ACEs. The final study employed latent class analysis (LCA) and subsequently utilized a three-step procedure to examine associations between classes and the outcome variable. The studies in this category focused on the impact on mental health and behavioral health outcomes. More specially, they tried to examine if including expanded ACEs can improve explanatory power of the model. Overall, the studies were of good quality. However, some of them did not use representative samples, which may limit the generalizability of the findings.

SmithBattle et al. conducted a comprehensive review of 18 ACE studies, encompassing individual items, aggregate ACE scores, and health-related outcome variables [62]. Bullying was assessed as a potential ACE in 10 studies. It was found that bullying was associated with an elevated risk for unfavorable mental health outcomes independent of other ACEs. In the context of physical health and psychosocial problems, no associations independent of original ACEs were observed. The strongest correlation was found between health outcomes and the total ACE score. Expanded ACE contributes to the ACE exposure of 14% and correlates as part of the total ACE score with many different health outcomes. Furthermore, they load on all relevant ACE categories that are consistent with the dose-dependent relationship established for traditional ACE and the theory of a burden, which builds up by experiencing ACEs [62].

A factor analysis was employed by both Karatekin and Hill, as well as Morrill et al., to ascertain the viability of incorporating expanded ACEs into the theoretical framework of ACEs [63,64]. The findings of both studies indicated the presence of a factor associated with bullying that contributed to the analysis. Karatekin and Hill stated that aversive experiences can also occur outside of the home and therefore are not captured by the original 10 ACEs [63]. Therefore, the objective of this study was to examine the potential expansion of the ACE scale. They build on the findings of previous research, which suggested different factor structures for the ACE concept. The sample population for this study comprised 1417 participants who were recruited from psychology classes at a public midwestern university. The sample was a convenience sample, which impairs the generalizability of the findings. Otherwise the study was of good quality. The researchers utilized the original ACE questionnaire in combination with the Juvenile Victimization Questionnaire (JVQ), resulting in a total of 33 items for the comprehensive assessment of ACEs. Mental and physical health outcomes, including anxiety, depression, and perceived stress, were measured as dependent variables. An exploratory factor analysis (EFA) was performed on half of the sample, followed by a confirmatory factor analysis (CFA) on the remaining half. The optimal model fit was obtained for a four-factor structure comprising child maltreatment, household dysfunction, community dysfunction, and peer dysfunction/property victimization. This model accounted for approximately 60% of the total variance in the data, and all factors exhibited strong intercorrelations [63].

Morrill et al. adopted a somewhat different methodological approach in their high-quality study [64]. Drawing on the existing literature, they conducted multilevel modeling analyses with a sample of 1302 participants and examined both micro- and macro-level processes through the inclusion of sibling data. Their model incorporated the original ACEs together with several additional indicators, such as bullying, with factors estimated at both the between- and within-family levels. Bullying was initially hypothesized to load on the “poor family environment” factor but was reassigned due to weak factor loadings. In the final model, bullying loaded at 0.59 on the “poor child environment” factor and demonstrated stronger criterion validity associations with adult psychological, social, and physical health outcomes than divorce, domestic violence, or overall family conflict. Inclusion of bullying substantially improved the overall model fit [64]. Both studies provide empirical support for the notion that a broader conceptualization of ACEs is psychometrically compatible with the original ACE framework and may yield improved predictive validity.

The examination of the extant literature revealed four studies that employed regression analyses as the primary analytic approach to examine the expanded ACE framework. Three of these studies employed psychological distress as the outcome variable [65,66,67], while one assessed alcohol use, cannabis use, externalizing and internalizing symptoms, and trauma [68]. Evidence was provided by all four studies for the individual contribution of bullying to mental health outcomes.

One of the earliest studies on the expansion of ACEs was conducted by Finkelhor et al. [65]. In the replication of the original ACE study, supplementary items from JVQ were incorporated, including peer victimization. The psychological distress experienced over the past month was assessed using the Trauma Symptoms Checklist for Children (TSCC). The findings indicated a dose-dependent relationship between ACE exposure and psychological distress. However, it is important to note that not all original ACEs demonstrated independent associations with distress in this study, such as divorce and incarceration. The regression model demonstrated a robust correlation between bullying and distress, thus providing substantiated evidence for the potential refinement of the ACE scale. However, study quality was impaired because of a considerable amount of dropout [65].

Sasaki et al. reported consistent findings [66]. The objective of their high-quality study with 28,617 participants was to expand the ACE framework to more effectively capture the lived experiences of the Japanese population. Consequently, bullying and other contextually relevant items, such as exposure to natural disasters, were incorporated into the study. The logistic regression model for psychological distress was adjusted for the following demographic and socioeconomic variables: age, sex, marital status, household income, employment status, and educational attainment. The prevalence of ACEs ranged from 3.2% for physical neglect to 38.5% for emotional neglect, with bullying reported by 20.8% of the participants, making it the third most prevalent ACE. All individual ACEs, with the exception of parental death, demonstrated a statistically significant association with elevated levels of psychological distress (odds ratios ranging from 1.23 to 4.01). These findings exhibited a dose-dependent pattern. School bullying has been demonstrated to be associated with a significantly elevated risk of psychological distress (OR = 3.04). The impact of bullying on mental health has been observed to be evident across all age groups [66].

Lampe et al. directed their attention to the original ACEs and peer abuse, conducting separate regression analyses for female and male participants [67]. The sample included 2307 patients recruited from a hospital setting. Since this is a convenience sample, generalizability of the findings is limited. Apart from that the study was of good quality. Psychological distress was measured using the Brief Symptom Inventory-18. The German Pain Questionnaire was also administered. A divergence in outcomes based on gender was observed. In the male sample, bullying was not identified as an independent risk factor for adverse health outcomes. However, a combination of peer abuse and other ACEs was associated with an elevated risk of depression, anxiety, somatization, and the presence of multiple comorbidity diagnoses. This finding aligns with the conclusion made by Finkelhor et al. that not all ACEs independently contribute to distress [65]. However, the inclusion of bullying further enhances the predictive validity within the dose-dependent ACE–distress relationship. Conversely, among the female population, bullying was found to be associated with an elevated risk of depression and somatization without other forms of ACEs present. Furthermore, the cumulative effect of ACEs and peer abuse was linked to an increased likelihood of somatization, comorbidities, depression, and anxiety [67].

Folk et al. sought to examine the relationship between expanded ACEs including bullying and risky health behaviors such as alcohol and cannabis use, as well as trauma, externalizing, and internalizing symptoms, in a sample of youths with first-time court involvement [68]. ACEs were highly prevalent in the sample. Approximately 90% of the participants reported at least one ACE, and 42% of them disclosed experiencing bullying. Contrary to prevailing assumptions, expanded ACE scores were not associated with behavioral health outcomes. However, they exhibited a substantial association with symptoms of trauma, internalizing, and externalizing symptoms. Notably, no correlation was identified between the original ACEs and trauma in this particular sample. However, the quality of the study might be limited because of various points. First, the sample is not representative, which limits the generalizability of the findings. Furthermore, there was considerable dropout, which might have introduced bias. Additionally it was not clearly reported in exposure might have changed during the study period [68].

Similar to the research question addressed by Folk et al., Smith et al. aimed to determinate the association between expanded ACE and cannabis use with a latent class analysis [69]. Since original ACEs are associated with substance use, it would be future evidence for including new ACEs if the same applies for extended ACEs [89]. The sample of the online survey consisted of 712 participants. The study was of good quality, but it was not reported how high the percentage of dropout was. Therefore no statement can be made regarding the generalizability of the findings [69]. Original ACEs were measured with exclusion of sexual abuse. An additional five extended ACEs were measured, encompassing community violence, discrimination, dating violence, foster care involvement and bullying. Cannabis use was measured by the time of consummation. In the event of any consummation during the previous six months, three items were utilized to assess problematic cannabis use. An LCA was employed. The identification of associations between the groups and cannabis use was accomplished in three steps. The study’s findings indicated that 63% of the participants had been exposed to at least one original ACE. Emotional abuse was identified as the most prevalent original ACE, accounting for 40% of cases. The prevalence of bullying was found to exceed the aforementioned figures, with 65% of the participants reporting its occurrence. Four classes were identified encompassing low ACE, interpersonal harm, interpersonal abuse and high ACE class. Bullying was prevalent in all classes, including the low ACE class. The elevated risk for cannabis use was observed to be significantly associated with the high ACE class, with an odds ratio of 6.2 in comparison to the low class. The study revealed no significant differences between the abuse and harm classes. However, they exhibited an enhanced risk of cannabis use compared to the low ACE class. In addition, an analysis was conducted to determine the risk of developing cannabis use disorder (CUD). The CUD score was found to be highest in the high ACE class; however, no significant differences were observed between the other three classes. A significant correlation has been identified between CUD and bullying exposure. This finding suggests that the inclusion of expanded ACEs in clinical assessments could enhance the accuracy of predicting health risks and should be considered in further research and clinical practice. Moreover, the findings indicate that bullying is not only prevalent among children with high ACE exposure, but it is also prevalent among children with low ACE exposure. Consequently, it may be more appropriate to characterize it as a distinct factor rather than as a form of revictimization [69].

Taken together, the studies identified within the expanded ACE category indicate that bullying exerts an additional influence on psychological distress, going beyond that explained by the original ACE framework. Although some inconsistencies remain regarding whether this influence operates independently or cumulatively, there is clear evidence that the inclusion of bullying enhances predictive accuracy. Moreover, several studies demonstrate that not all original ACEs consistently display independent associations with outcome variables, yet they collectively contribute to the overall impact of ACEs on mental health. It is important to note that all these studies focused on mental health outcomes. Future research should aim to replicate these methodologies in relation to physical health outcomes, which are also known to be significantly influenced by ACE exposure.

#### 3.1.2. Associations Between ACEs and Bullying

Three of the identified studies focused on the question of whether ACEs are a risk factor for bullying perpetration and victimization [70,71,72]. This area is of interest, since one ACE can increase the risk for other ACEs [90]. The first study is a literature review synthesizing research conducted over the previous two decades [70]. Nagata et al. specifically researched the phenomenon of cyberbullying and Sapouna et al. conducted a study on a Scottish birth cohort to seek further evidence, as suggested by Merrin et al. [71,72]. In their literature review, Merrin et al. found overall positive correlations between bullying perpetration and cumulative ACE score, maltreatment, physical abuse, family violence, and household violence. Mixed results were identified for sexual abuse, emotional abuse, substance use in the household, mental illness of a caregiver, and separation of the parents.

Regarding bullying victimization, positive correlations were identified for cumulative ACEs, physical abuse, emotional abuse, household violence, maltreatment, family violence, and neglect. The results for sexual abuse and separation of parents were inconclusive, similar to the findings for the perpetration data [70].

Sapouna et al. conducted a study on a sample from the GUS study [71]. The sample population comprised 2669 children born between June 2004 and May 2005 in Scotland. The longitudinal study consisted of 10 sweeps and spanned 14 years of the young people’s lives at that time. The outcome data is incomplete, since a considerable number of dropouts were observed. This may have led to biased results. Furthermore, it is not clearly reported if exposure changed during the study. Eight ACEs were measured in the study; emotional abuse and physical neglect were not assessed. Notably, sexual abuse and incarceration of a caregiver were excluded from the multivariable logistic regression analysis due to their minimal prevalence in the sample. The cut-off for high ACE exposure (typically four or more) was set to three or more. One-third of the sample reported experiencing bullying victimization, making it almost as prevalent as separated or divorced parents, which was the most common ACE (37%). The analysis was controlled for sex, ethnicity, and SIMD area quintile, which provides a relative rating of deprivation zones in Scotland. In the context of bullying perpetration, substance misuse emerged as the sole significant predictor, although this association did not persist in the multivariate analysis. In the subgroup analysis consisting of females only, separation or divorce of parents and a high number of ACEs experienced predicted the risk of perpetrator ship. In the multivariable model, only divorce or separation remained a predictor. The only significant predictor for bullying victimization in the entire sample was parental mental health problems; however, this predictor lost its significance in the multivariate analysis. In the analysis limited to female subjects, substance misuse and parental mental health problems predicted victimization. Furthermore, the presence of one ACE has been demonstrated to elevate the risk of becoming a victim of bullying. In the multivariate analysis, only substance use remained significant. It was discussed that ACE exposure could be linked to bullying involvement through emerging externalizing (perpetrators) and internalizing (victims) problems. The results were consistent with those reported by Merrin et al. in terms of heterogeneity between the ACEs. However, surprisingly, different ACEs were linked to bullying perpetration and victimization. The authors posited that the observed discrepancy could be attributed to the utilization of disparate methodologies. This study was not conducted retrospectively, and the children were only 14 years of age. Notably, a gender difference was observed [71].

Nagata et al. sought to address the relationship between ACEs and cyberbullying in their high-quality study [72]. They assessed eight ACEs, excluding incarceration and emotional abuse, in a sample of 10,317 children as part of the ABCE study. The mean age of participants was 12 years. The researchers identified a dose–response relationship between the number of experienced ACEs and cyberbullying victimization. Furthermore, sexual abuse, household mental health problems, substance abuse in the household, and household physical abuse have been found to be significantly associated with cyberbullying victimization. Among the variables examined, sexual abuse emerged as the strongest predictor of adverse outcomes. Notably, children who had experienced ACEs exhibited a tendency to engage in more extensive screen time in comparison to their peers. The authors of the study posited that the subjects’ elevated risk of cyberbullying can be attributed to both the subjects’ changed behavior and the lack of control exercised by their caregivers. Furthermore, the finding that sexual abuse is the strongest predictor aligns with prior research, underscoring the heightened vulnerability of victims of sexual abuse and the risk of subsequent victimization [72].

Taken together, these studies all show support for a link between bullying and ACE exposure. Two of the studies involved children and are therefore comparable. However, they differ in the type of bullying examined. In general, cyberbullying is less common than in-person bullying and might therefore affect a more specific group of victims.

However, the link between ACEs and bullying underscores the possibility that bullying may be a form of revictimization, as the “circle of violence” theory proposes [91]. The results suggest that exposure to ACEs is a risk factor for bullying and align with the revictimization theory.

This raises the question of whether bullying contributes sufficiently to negative health outcomes to be acknowledged as a distinct form of ACE. It is important to note that experiencing one form of ACE increases the likelihood of experiencing other forms [90,92]. The results of the previously discussed studies should not be viewed strictly as evidence against the hypothesis that bullying is an ACE, but rather as an alternative explanation of the connection.

#### 3.1.3. Latent Class Analysis

Three of the identified studies approached the topic of bullying and adverse childhood experiences (ACEs) using LCA. LCA is a methodological approach that is person-centered rather than variable centered. The majority of previous research on ACEs has employed a variable-centered approach, which utilizes a cumulative score to examine associations with different health outcomes. In light of the established dose-dependent relationship, this methodological approach appears to be a rational one. Nevertheless, LCA provides novel opportunities for comprehending the dynamics between ACEs and bullying. LCA enables researchers to account for the varying impacts of individual ACEs on participants’ health and the differential effects of distinct ACE clusters by identifying subgroups of ACEs [93,94].

The reported studies incorporated bullying as an ACE and sought to identify ACE classes. The assessed outcomes included long-term mental and physical health outcomes, as well as behavioral health outcomes, and therefore cover a wide range of outcomes observed in association with ACEs. All studies identified a high- and a low-adversity class, as well as a class consisting of bullying and emotional abuse or neglect, despite variations in the nomenclature. Therefore, there is substantial evidence supporting the conceptualization of these classes of ACEs. One study additionally identified a poverty class, which may be due to the inclusion of a broader set of ACEs.

All studies found evidence for a dose-dependent relationship, as the high or multiple adversity classes exhibited a heightened association with adverse health outcomes, thereby aligning with extant findings [73,74,75]. However, a number of slight discrepancies were observed between the studies. Bond et al. found in their study of 729 students that bullying had the highest endorsement among all ACEs measured (including parental mental illness, substance misuse, physical, emotional, and sexual abuse, neglect, parental violence, and criminal behavior, along with additional questions exploring these topics in depth), with 20% [73]. Their bullying class was characterized by particularly high levels of parental insults and verbal and exclusionary bullying, accompanied by moderate levels of parental mental illness, parental substance misuse, and physical violence. The high-ACE class demonstrated elevated levels across all adversities, with the exception of parental suicide.

Particularly interesting is that even the low-ACE class experienced moderate levels of bullying. This indicates that bullying is prevalent across all classes and not solely a phenomenon experienced by individuals with high ACE scores and the highest vulnerabilities. Moreover, the bullying class exhibited higher levels of depression and suicidal ideation compared to the high-ACE class, suggesting that bullying, in conjunction with emotional violence in the domestic environment, poses a heightened risk for children. Bond et al. also noted that a prior class analysis using the same dataset had excluded bullying. Their results demonstrated that bullying improved the explanatory power of the model, providing strong evidence for its inclusion as an ACE. However, the handling of potential confounders was not clearly described, limiting the ability to assess the risk of confounding bias [73].

Liu et al. as well as Hirai et al. did not concentrate specifically on bullying in their studies [74,75]. However, instruments that included bullying as a factor were utilized, and the findings were consistent with the previous reported study.

Liu et al. employed the ACE-IQ in a study of 4441 participants living in Singapore [74]. The study was of good quality, however, there was a considerable dropout rate, which may have introduced bias. As with previous studies, they assessed mental health, physical health, and risky behaviors such as smoking and binge drinking. However, the scope of their research was expanded by examining the moderating and potentially protective effects of social support and positive mental health. Consistent with the findings of the preceding two studies, they identified an “emotionally neglected and bullied” class, characterized by high levels of emotional neglect and moderate levels of bullying. It was determined that social support and positive mental health indeed moderated the effects of bullying. Social support could buffer the impact of ACEs to such an extent that no significant difference in suicidality was observed between the low- and high-ACE classes, underlining the power social factors exhibit over health outcomes [74].

Hirai et al. assessed the original ACEs, excluding parental incarceration but including parental death, financial hardship, lack of respect for opinions, illness or hospitalization, bullying, and natural disasters, in a representative sample of 28,042 Japanese adults in their high-quality study [75]. Their outcome variables included physical health, mental health, and a category designated as “health/abuse,” which comprised smoking, drinking, and both physical and psychological abuse toward others. They found three classes corresponding to the other two studies and also identified an additional “poverty” class, resulting in a total of four distinct ACE patterns. Their “psychological abuse” class was consistent with the bullying class identified by Bond et al., as bullying was highly prevalent in this group. All classes had negative effects on physical health, mental health, and abuse-related behaviors. The findings indicated that all classes exerted adverse effects on physical health, mental health, and abuse-related behaviors. While the multiple adversity class exhibited the highest risk, supporting the dose-dependent relationship, the study also demonstrated that even experiencing a few ACEs can have long-lasting impacts [75].

These studies provide substantial evidence regarding whether bullying should be considered an ACE, as it was prevalent and impactful enough to form a distinct class in all three studies. Moreover, the research suggests that bullying especially affects children when combined with emotional forms of ACEs at home. Therefore, it has not only a statistically significant impact on predicting mental, physical and behavioral health outcomes but also practical clinical implications for identifying particularly vulnerable children. Additionally, bullying was prevalent in the “middle” class between low and multiple ACE classes across all studies, and in some cases even in the low-ACE class [73,74,75]. This provides further evidence that it may not be sufficient to view bullying solely as a form of revictimization. Instead, there appears to be a place for bullying within the ACE framework.

#### 3.1.4. Biological Stress Markers

Several studies researched the biological embodiment of ACEs and bullying. A total of three studies were identified for this review, which allows for a direct comparison between a substantial number of original ACEs and bullying. The topic of biological stress markers is of interest, given that the prior association of various long-term consequences on physiological systems were previously connected to ACEs. Similarities in biological embodiment could therefore support the hypothesis that bullying should be considered another ACE. The first study was conducted on DNA methylation in young individuals [76]. The longitudinal study comprised a sample of 2232 twins and assessed physical, sexual and emotional abuse, neglect, domestic violence, bullying, cyberbullying and crime. However, no consistent or reproducible associations were identified for cumulative scores of victimizations or any specific form of victimization. Outcome data was incomplete for this study, which may have led to biased data. However, the study performed good in other quality assessment categories [76].

Soares et al. conducted a study on 8647 mothers and children to explore the relationship between inflammation and BMI in 10-year-old children [77]. The study was of good quality; however, the extent of dropout was not clearly reported. Therefore, it is unclear whether the results may be biased. ACEs and bullying were assessed and Pearson correlations as well as path analysis were conducted. A positive yet weak correlation was identified between BMI and the inflammation marker, high-sensitivity C-reactive protein (hs CRT). A divergence of outcomes was identified, contingent upon the nature of the effect in question. The impact on hs CRP was predominantly direct for ACEs and predominantly indirect for BMI in cases of bullying. This finding is noteworthy as it demonstrates variations in the embodiment of bullying and other forms of ACEs. This phenomenon may be attributed to the tendency of affected individuals to engage in stress eating as a coping mechanism in the context of bullying. This finding suggests that the negative effects of bullying may emerge on a different path than the original ACE and, therefore, could be categorized differently [77].

Iob et al. conducted a high-quality rated longitudinal cohort study on the severity levels of inflammation and depression throughout childhood in relation to ACEs [78]. The sample under consideration consisted of 3931 children from the ALSPAC study. A comprehensive set of factors was measured across the life course, from prenatal to adolescence, including physical, emotional, and sexual abuse; household violence; parental substance use problems; parental mental health problems; parental convictions; parental separation; low parent–child bonding; and bullying. The statistical analysis was performed with EFA and CFA to form groups of ACEs. Two distinct groups were identified: a physical/emotional threat group and a household dysfunction group. It is noteworthy that sexual abuse and bullying did not integrate with the two identified ACE groups. Instead, they were considered as standalone factors. LCA was employed to establish severity groups for CRP trajectory and depression. The study identified three severity groups of depression: low, moderate, and severe. Concurrently, three groups of CRP levels were identified. The present study employed multinomial logistic regression to assess the associations between ACEs and depression, as well as inflammation. A notable finding of the study was the exclusive association of elevated CRP levels with bullying and sexual abuse among male subjects. It was posited by the authors that these experiences occur during the period of late childhood. In accordance with these findings, higher levels of C-reactive protein (CRP) were observed in the household dysfunction group among adolescents. However, no significant differences in CRP levels were detected in response to individual ACEs within this group. Exposure to ACEs has been demonstrated to be associated with moderate and severe depression. However, no such association has been identified between inflammation and depression severity. In light of these findings, bullying is distinguished from the other ACEs in two distinct ways. First, it does not align with either of the two groups identified in the factor analysis. Second, it is associated with inflammation, a characteristic that other single ACEs, with the exception of sexual abuse among male participants, do not exhibit. This finding suggests the potential for a discrepancy in the mechanism of embodiment, which could serve as an indication that bullying does not align with the other ACEs. However, it is important to note that sexual abuse also does not align with any of the identified groups in this study and is undoubtedly an ACE. Furthermore, enhanced CRP levels were observed for sexually abused boys. It was hypothesized that these enhancements could be indicative of a subsequent exposure time, due to the fact that both experiences tend to manifest during later childhood or adolescence. Moreover, bullying is an experience that generally transpires in school settings or during leisure time, rather than within the confines of the family unit. Although sexual abuse can occur within the family unit, it is important to note that it is not limited to the parents. It is, in fact, an extreme and rare form of child abuse when compared to the other ACEs. Therefore, there is a higher probability for other ACEs to coalesce, which could provide a rationale for the isolation of these factors [78].

In summary, the three studies on biological stress markers, more specially CRP and DNA methylation selected for this review, offer only limited support for the hypothesis that bullying should be considered an ACE. Furthermore, the results of two studies suggest the presence of alternative modes of embodiment, which could imply that bullying may not be adequately characterized as an additional ACE. It is noteworthy that all studies included in this category have one element in common: the samples are composed exclusively of young individuals. Studies on ACEs frequently utilize retrospective measurements, a methodological approach that has the potential to yield divergent outcomes. However, a discrepancy between ACEs and bullying can be observed regarding biological stress markers. Further research is necessary to investigate this discrepancy, as the nature of the difference may be significant. The premise of bullying being an ACE would not be strongly contradicted by a difference in the time of experience. Conversely, a discrepancy in the nature of the experience would suggest an alternative interpretation. A notable consideration is the association between bullying and CRP, which is distinct from the associations observed between other ACEs, excluding sexual abuse, and CRP. Consequently, it is a distinction, yet not an indicator absent in bullying, while it is noted in other ACEs in this particular study. Therefore, it can be hypothesized that bullying is a particularly damaging experience, which would suggest that it exerts a more significant influence on CRP levels. The disparities must be considered in the discourse surrounding ACEs and bullying. Nevertheless, given the paucity of evidence from the two extant studies, it is not possible to conclude definitively that bullying is not an ACE.

#### 3.1.5. Negative Health Outcomes Associated with ACEs and Bullying

Eleven of the identified studies examined the association between ACEs and adverse health outcomes and included bullying. The studies included in this category can be further subdivided into physical health consequences that persist into adulthood and studies on mental health consequences. A number of the included studies were conducted on adults and, as a result, reported on long-term consequences. Other studies were conducted on children, thereby illustrating more immediate consequences of ACEs and bullying. The quality of the studies included in this section is mixed. The most common problems encountered pertain to the incompleteness of outcome data and the non-representativeness of the sample. Three of these studies focused on physical health issues like diabetes, pain levels and chronic diseases [52,79,80]. A study of 30,403 Icelandic woman revealed a strong correlation between bullying and diabetes type 2. This association was further substantiated in a second model that controlled for other ACEs and adult BMI, with bullying emerging as the strongest predictor, followed by sexual abuse and mental illness of a household member [79]. Moreover, it was found that ACE exposure predicts increased risk of diabetes type 2 by approximately 10% for each additional ACE. It was hypothesized that the observed association may be attributed to elevated HPA-axis activity, subsequently resulting in elevated blood glucose levels. The study was of good quality, but a considerable dropout rate was observed, which may have biased the results [79]. Another study examined the correlation between pain levels and ACEs in an adult sample [52]. While the study was of good quality, the outcome data was not complete. The strongest correlation was identified with emotional abuse. A moderate correlation was identified between bullying and “any pain,” indicating a stronger association compared to sexual abuse and both forms of neglect. In the “bodily pain” category, the association with bullying was found to be comparable to that of physical neglect. In this case, bullying exhibited one of the least strong correlations when compared to other ACEs. In the category designated as “headache,” bullying exhibited the third strongest correlation, following emotional and sexual abuse. The findings of this study underscore the observation that individual ACEs demonstrate varying degrees of correlation with disparate outcomes. It is important to note that not all ACEs exhibit the same correlation with the outcomes. For example, an ACE with a weak correlation with one outcome can have a strong one in a different category [52]. Another study conducted with 454 patients in the South Bronx measured correlations between chronical illnesses and ACEs using multiple logistic regression [80]. The sample was a convenience sample, which limits the generalizability of the findings. Additionally, study quality is limited by the incomplete outcome data, which might have introduced bias. Seven original ACEs were measured in conjunction with several other stressful childhood experiences including bullying. A correlation has been demonstrated between several ACEs and the development of chronic diseases. A significant correlation was identified between bullying and one chronic disease (OR 4.84), which is comparable to the associations with separation or divorce of parents. Bullying exhibited the strongest correlation after sexual abuse (OR 21.53). Another significant correlation was identified between having three chronic diseases and bullying (OR 2.00). In this case, the effect size was commensurate with that observed in the context of substance abuse, mental illness in the household and separation or divorce of parents. There was no significant correlation between two chronic illnesses and bullying (OR 1.7), though the effect size was comparable to that of incarceration of a household member and substance use of a household member. An association between bullying and hypertension, hyperlipidemia and dysglycemia was revealed. Analogous correlations were identified for sexual abuse and mental illness in the family [80].

These studies show that bullying integrates well with the other ACEs regarding physical consequences and exhibits adequate predictive power. Furthermore, the strong correlations between bullying and adverse health outcomes are well-documented, underscoring its significant impact.

A series of studies including bullying as an ACE were conducted on the impact on mental health. A recent study has indicated a correlation between the earlier onset of borderline personality disorder and adverse childhood experiences, such as abuse, neglect, household dysfunction, and bullying [81]. This study employed a convenience sample but was of good quality otherwise. The earlier onset has been demonstrated to be connected with diminished social functioning [81]. De Vries et al. conducted a study on emotional and behavioral problems and their connection to ACEs [82]. It was not clearly reported how large the dropout rate was. Additionally, it is uncertain whether there were changes in ACE exposure during the study period. These two points might limit study quality. Six original ACEs were included alongside bullying. The sample consisted of a cohort study with 1880 participants, with measurements taken at 12, 14, and 20 years. The measurement of ACEs was conducted until the subject reached the age of 14 (or 16 for cases of sexual abuse). A direct correlation has been demonstrated between the presence of specific ACE factors and an elevated risk of emotional problems, including victimization by bullying, peer rejection, parental mental health issues, emotional abuse, and sexual abuse. The only ACEs directly associated with an increased risk of behavioral problems were emotional abuse and parental divorce. This study employed a novel approach and investigated the impact of individual ACEs as well as associations between ACEs to differentiate between direct and indirect effects through community detection. This approach demonstrated greater stability in terms of confounding. Bullying was identified as the sole ACE that was grouped with emotional problems and remained a relevant factor when controlled for exposure to other ACEs. Another result was that the original ACEs are not a homogeneous construct; rather, they exhibit differential impacts. This finding suggests a need for greater emphasis on adverse experiences associated with peer groups. Furthermore, the evidence suggests that bullying exerts an impact that extends beyond the scope of other ACEs. This finding lends further credence to the notion that bullying should be regarded as a distinct ACE. In the event that the phenomenon under investigation manifested solely as a form of revictimization, it would have been anticipated that it would cluster with other ACE factors, as opposed to aligning with the outcomes observed in the community analyses [82]. Another study was conducted with 1062 French college students to investigate the relationship between attention deficit hyperactivity disorder (ADHD) and ACEs [83]. The study was rated as high-quality. It was found that each ACE elevates the risk for ADHD. The strongest associations were identified for sexual abuse, both forms of neglect and bullying (aOR 2.5). It was hypothesized that the risk of developing ADHD is elevated due to the repercussions of toxic stress, particularly in conjunction with genetic predispositions [83]. Laporte et al. conducted a high-quality rated study on the correlation between self-harm and ACEs in 98 forensic patients [84]. The measurement of ACEs was conducted using the ACE-IQ, complemented by supplementary interviews. The findings of the study indicated a positive correlation between the presence of each additional ACE and an increased risk of self-harm. The presence of ACEs was found to be more prevalent among the subgroup exhibiting symptoms of self-harm and suicidality. The odds ratio between bullying and self-harm was not among the highest observed; however, it was found to be more pronounced than the odds ratio associated with sexual abuse and absence of a parent. In consideration of the subject’s suicidality, bullying exhibited an impact that was commensurate with that of parental substance misuse. Furthermore, the impact of bullying surpassed that of household violence, parental mental illness, and abuse. It should be noted that a direct comparison of the employed Childhood Trauma Questionnaire measurements was not possible due to the utilization of divergent analytical methods. The only ACEs that demonstrated a substantial correlation with both self-harm and suicidality were emotional abuse and emotional neglect [84]. Trompeter et al. conducted a study on 8085 children to illuminate the overlap between bullying and ACEs regarding internalizing and externalizing problems [85]. In this study, cyberbullying and in person bullying were measured. While the study was of good quality, a considerable dropout rate was observed. The results suggest that both bullying and ACEs independently contribute to externalizing and internalizing problems. The anticipated relationship, characterized by its dose-dependent nature, has been demonstrated. Nonetheless, discrepancies were identified in the association between individual ACEs and the outcome variables. For instance, physical neglect was found to be associated exclusively with externalizing problems, while emotional neglect, physical abuse, and parental divorce were observed to be unrelated to internalizing or externalizing problems. It was discussed that screening for both ACEs and bullying should be employed, as the risk for externalizing problems increases when both factors are present. Furthermore, the findings indicated a high probability of cooccurrence between bullying and ACEs [85].

A study was conducted by Manoli et al. that examined the relationship between bullying and maltreatment during childhood and the clinical expression of bipolar disorder [86]. The sample comprised 63 individuals with a confirmed bipolar disorder diagnosis who participated in a 10-year follow-up of the BADGE study. The study was of good quality but it is not clearly stated if the sample is representative and therefore generalizability of the results might be impaired. Five types of maltreatment were assessed retrospectively for the purpose of ACE measurement. Bullying occurrence was measured retrospectively between ages of 4 and 16 years. In this study, participants were divided into two groups: those who had been subjected to bullying and those who had not. These groups were then analyzed statistically to determine any differences. The results of the study indicated a correlation between bullying and worse clinical expressions of bipolar disorder, manifesting in the form of heightened suicidality and the presence of psychotic symptoms. Following the incorporation of maltreatment as a covariate, the association with suicidality persisted as a significant outcome. A comparison was made between groups that had been exposed to only one form of adversity (i.e., bullying or maltreatment) and those who had been subjected to both forms. The results indicated that the group that had been exposed to both forms exhibited a significantly higher prevalence of suicidal behavior. This finding suggests an additive relationship between the two forms of adversity, emphasizing the necessity to broaden the understanding of ACEs [86].

Wakuta et al. conducted a high-quality rated study examining the depression risk associated with the Japanese phenomenon of hikikomori, a condition that is resulting in significant socioeconomic costs [87]. Hikikomori is a psycho-sociological condition that was first studied in young Japanese individuals. It is characterized by a severe social withdrawal that has persisted for a minimum of six months. Affected individuals primarily remain at home, do not engage in school, work or social activities and do not have close friends. The condition manifests in the absence of a psychotic disorder or a noticeable intellectual disability [95]. In this study, the school ACE was measured alongside the original ACE, enabling a direct comparison between the two. The items that were enabled in School Ace were grouped into two categories: teacher-related items and bully-related items. The former category included emotional, physical, and sexual abuse, as well as instances of violence against friends. The latter category included bullying by classmates and older students. A total of 4000 Japanese adults participated in the online survey. While 35% of their participants experienced at least one ACE, 55% experienced at least one school ACE and 50% responded positively to at least one bullying item. For each ACE the risk of depression increased by 24%. For school ACE the risk of depression increased by 44% per ACE, which is notably higher than the increase identified for original ACE in this study. This finding underscores the importance of adverse school experiences on subsequent mental health outcomes, which were also reported for original ACEs. For hikikomori, an elevated risk was identified solely for school-based ACEs (29%), while no such elevated risk was observed for original ACEs. The prevalence of risk for hikikomori was observed to be most significant among individuals who had previously experienced bullying, with an elevated risk of 37%. In addition, it was determined that 71% of the children who experienced original ACEs were exposed to at least one school-related ACE. This finding indicates that Japanese children encounter elevated levels of adversity within the academic setting, surpassing that experienced in their familial environments, with notable consequences for their mental health. This study’s findings provide additional support for the necessity of expanding the scope of ACEs [87].

Miller et al. conducted a study to compare the impact of bullying on emotional distress in adulthood with the impact ACEs have on emotional distress [88]. The study rated good in terms of quality. However, it is uncertain if the sample can be considered representative. Because of this, results might have limited generalizability. In addition, the objective was to ascertain whether the stress response is cumulative and to elicit a ranking of their experiences according to adversity from the participants. It was asserted that bullying constitutes a form of trauma in accordance with the criteria established by the SAMSHA (Substance Abuse and Mental Health Services Administration) definition. In a sample of over 500 participants, it was revealed that 28% of the participants identified bullying as their most aversive experience. These results indicate that approximately one-third of the respondents reported experiencing a profoundly detrimental impact on their lives because of bullying, underscoring the gravity of this phenomenon. The path analysis was significant for both bullying and cyberbullying in females and only for cyberbullying in males. For both genders, an interaction of bullying and ACEs was found. This finding provides further support for the inclusion of bullying as an ACE, given the numerous commonalities between ACEs and bullying. The hypothesis proposed that the gender disparity observed was attributable to a heightened negative response exhibited by females. Nonetheless, the evidence is limited due to the fact that the study is not longitudinal. This limitation stems from the possibility that stress may be confounded by stressful life events that have occurred more recently. Consequently, causal attributions are not possible [88].

The extant literature suggests a positive correlation between bullying and negative mental and physical health outcomes linked to original ACEs. Those correlations are often comparable to those of original ACEs. Moreover, studies show that bullying can have a particular detrimental effect on affected individuals, for example the impression that bullying was the worst experience or becoming a hikikomori. A brief overview of the key findings for all studies is presented in Table 2.

### 3.2. Bullying as an Adverse Childhood Experience

It has been postulated that there is an increasing body of evidence indicating the potential convergence of bullying with other ACEs in regard to mental, physical and behavior health consequences, which were previously associated with original ACEs. To provide a more comprehensive context for the results described, this section will first examine the historical development of the concepts of bullying and ACEs, following stage 5 of the methodology described by Barry et al. [60]. The conclusions drawn in the previous part will undergo a process of integration and comparison with the results of other research studies, with the aim to provide a compressive overview over the state-of-the-art understanding regarding whether bullying is an ACE. Following the initial ACE study, the concept of ACE has undergone significant development, with numerous recommendations proposing the inclusion of bullying, community violence, and poverty as additional components. Recently, there has been a movement to focus more attention on bullying as a possible ACE, beginning with Finkelhors’ study on extended ACEs [65]. Another significant milestone that contributed to the incorporation of bullying in ACE studies was the WHO’s formal acknowledgment of bullying as an ACE, thereby leading to its inclusion in the ACE-IQ [96]. Nevertheless, the concept of ACEs is employed differently by most authors, who include new ACEs and exclude old ones. A growing body of studies has emerged that includes bullying as a contributing factor in their total ACE score. However, the varied conceptualizations and applications of ACEs complicate the comparison of study results, as related yet distinct concepts are utilized under the same terminology. Nevertheless, measures were implemented to formalize ACEs through the validation of the ACE-IQ, which encompasses bullying. The evidence examined in this review paper demonstrates a high degree of similarity between bullying and original ACEs in numerous respects. It is evident that both ACE research and research on bullying have undergone significant development. Initially, bullying gained prominence within the domain of social science, with a significant focus on educational systems and preventive measures. Olweus’s initial and enduring definition has served as the foundational framework for understanding the concept to this day [11].

In recent years, there has been an increasing recognition of bullying as a traumatic experience [97,98,99,100,101]. Nielsen et al. concluded that the symptoms of posttraumatic stress disorder (PTSD) and those of individuals that were exposed to bullying are comparable [97]. The researchers identified correlations between bullying and PTSD symptom scores of 0.42 in both children and adults. Comparable significant correlations were identified between symptom intrusion, hyperarousal and avoidance with bullying [97]. A review conducted by Idsoe et al. established an association between bullying and trauma, finding that 30–40% of children exposed to bullying exhibited PTSD symptoms that exceeded the clinical cut-off [98]. Ranney reported that over 60% of the children that had a Child PTSD Symptom Scale score over 11, indicating a positive PTSD diagnosis, were exposed to bullying. Over 60% of the sample reported being a victim of cyberbullying. The sample was drawn at a Level I trauma center’s pediatric emergency department [102]. However, the question of whether bullying can meet the criterion of experiencing a life-threatening event remains unresolved. Bullying victims frequently report experiencing fear for their life [98]. Additionally, bullying is a recurrent phenomenon that occurs over an extended period of time and is nearly impossible to escape. Repeated exposure to bullying by peers has been demonstrated to result in significant alterations in the stress response of children [103]. Both Jenkins et al. and Lydecker propose the conceptualization of bullying as a traumatic event and advocate for a specialized approach to its treatment, such as the CBITS (Cognitive Behavioral Intervention for Trauma in Schools) or trauma focused cognitive behavior therapy. Furthermore, it was noted that “ACEs” and “trauma” are frequently used as synonyms. This observation lends further credence to the hypothesis that bullying may be classified as an ACE, given the established recognition of bullying as a traumatic experience [99,100].

In their recent study, Noret et al. sought to ascertain the efficacy of the trauma-focused approach in treating children who have been subjected to bullying. A total of 18 patients participated in a 12-week cognitive behavioral therapy (CBT) program. A substantial improvement in symptoms was observed following treatment [101]. Frewen et al. conducted an online study, which included 418 participants, investigated the relationship between PTSD, ACEs, traumatic events in adulthood, and stressful but non-traumatic events [104]. The investigation revealed that both traumatic events and ACEs were capable of predicting traumatic symptoms. However, it was also observed that stressful but non-traumatic events did not possess the same predictive capacity. Complex PTSD was predicted solely by the occurrence of ACEs. It was concluded that this evidence contradicts the premise that stressful but non-traumatic events can lead to PTSD symptoms [104]. Moreover, the synthesis of the aforementioned studies suggests that bullying should be classified as a traumatic experience, since it is associated with trauma symptoms. Given that it occurred in childhood, it might be considered an experience that fits well into the ACE model. Nevertheless, given that revictimization by peers or parents is a prevalent occurrence, it is imperative that subsequent studies incorporate measures to control for this. Recently, there has been an increased focus on the issue of cyberbullying. Research has indicated that cyberbullying can lead to symptoms of trauma as well [105]. Furthermore, the objective of the study was to ascertain which form of cyberbullying is most detrimental. Surprisingly it was revealed that all forms of cyberbullying, including threats of violence, exclusion, indirect cyberbullying, privacy violation and identity-based cyberbullying are equally harmful. A positive correlation was identified between the extent of cyberbullying experienced by individuals and the subsequent reporting of trauma symptoms. This suggests that the salient issue is not the nature of cyberbullying, but rather the extent to which it is experienced. In light of the profound and detrimental impact of cyberbullying, there is a growing discourse surrounding the potential inclusion of this phenomenon within the broader conceptualization of ACEs [105]. Given the similarity it shares with face-to-face bullying, this further underscores the gravity of all forms of bullying as a traumatic experience.

The trauma approach has raised attention to both the prevention and treatment of children who have been victimized by peers. In their review, Lyons et al. included bullying as an ACE to ascertain the developmental trajectory of ACE prevalence over time [106]. The most substantial increase was found for bullying, from 23 to 47%. Other factors that increased were household mental illness and discrimination. Concurrently, there was an increase in other factors, including household mental illness and discrimination. It has been demonstrated that bullying has a demonstrable impact on an increasing number of children, thereby contributing to an escalation in their cumulative exposure to ACEs, if included in the ACE model. This is of particular significance, as research has demonstrated that children who have experienced four or more ACE events are subsequently at heightened risk for developing health complications. Moreover, a dose-dependent relationship has been observed between the number of ACEs and health issues. The ACEs list was identified as a potential area for enhancement [106]. In light of these findings, what conclusions can be drawn from the extant literature on ACEs and bullying?

The studies in the category “expanded ACE” demonstrated that bullying does elevate the risk for mental health outcomes. The majority of studies have shown both individual associations to health outcomes and a contribution to dose-dependent outcomes, just as original ACE studies have shown [62,65,66]. This observation is consistent with other findings in the field. In a study conducted in Chile, the ACE-IQ was employed as an ACE measurement. In this setting, the most significant predictors of mental health conditions were found to be sexual abuse, bullying, and exposure to collective violence. Bullying was associated with depression, general anxiety disorder, suicidal ideation, self-harm and learning disorder. Furthermore, the odds ratio for PTSD was found to be higher in cases of bullying than in cases of sexual abuse. These findings underscore the meaningful contribution of bullying as an ACE in identifying vulnerable groups and underlines similarities between bullying and ACEs regarding long-term mental health outcomes [107]. A contribution of bullying victimization in conjunction with ACE exposure has been demonstrated with respect to the risk of traumatic brain injuries, which are defined as acquired brain injuries that may be occasioned by traumas to the head, such as blows and hits, and which have the potential to result in brain damage or interference with normal functioning and could therefore lead to the area of physical health outcomes. The cooccurrence of both factors raised the risk of suffering an traumatic brain injury significantly compared to exposure to ACEs without bullying involvement [108]. Two separate factor analyses reviewed in this paper demonstrated the presence of a factor for peer victimization that enhanced the overall model, thereby indicating an independent contribution [63,64]. A subsequent study identified a two-factor solution, with the first factor designated as “child maltreatment and peer victimization” and the second factor labeled “household challenges.” Furthermore, all ACEs included in this study were associated with poor self-rated physical or mental health outcomes. As indicated by the findings of previously reviewed studies, this finding lends further support to the incorporation of expanded ACEs and the conceptualization of bullying as an ACE. This is due to the fact that bullying demonstrates a factor loading and enhances the model [109].

The sole outcome contradicting the premise that bullying is an ACE is the lack of association between expanded ACEs and behavioral health outcomes, an area frequently linked to ACEs in previous research [68]. In addition to the aforementioned study by Smith et al. [69], a few other studies in the field linked ACEs to behavioral health outcomes and might help to provide a better understanding of this topic. An association was identified between items classified as “Household dysfunction ACE” and smoking, alcohol dependency and obesity, as well as with lower education and income [110]. Furthermore, an elevated risk of substance misuse and disorder has been associated with ACEs, as highlighted in a recent umbrella review [111]. Another study explored the relationship between different forms of victimization and early substance misuse [112]. The findings of the study indicated that both bully victimization and physical abuse by parents predict early substance misuse. In addition, cognitive impulsivity has been demonstrated to play a mediating role [112]. A study involving Australian adolescents revealed elevated levels of substance use among victims of bullying, though this was not observed among those categorized as passive victims [113]. Two studies indicate that bully victimization might be linked to elevated e-cigarette use in students [34,114]. One study assessed the association between a collection of factors that have been labeled as ACEs, bullying and substance use. It is important to acknowledge that the ACEs evaluated in this study were not the original ACEs but exhibited some degree of overlap. Both ACEs and peer victimization were associated with increased substance use. Furthermore, the analysis revealed a cumulative effect for experiencing both ACEs and bullying [115]. The findings of these studies contradict those of the study conducted by Folk et al., which found no associations. This discrepancy suggests the need for further research in this area.

Studies included in the category “associations between ACEs and bullying” examined whether ACEs function as a risk factor for bullying. Overall, the reviewed studies identified a positive correlation, thereby suggesting that children who have experienced ACEs are at a greater risk of being bullied or bullying others [70,71,72]. Furthermore, research has demonstrated that increased exposure to ACEs has been associated with an elevated risk of victimization, perpetration, or a combination of victimization and perpetration [116]. The association between ACEs and victimization exhibited variability across gender and analytical approach, as well as study. A recent study was conducted on the association between ACEs and bullying that could enhance the understanding of this observation made across the reviewed studies. It was found that the association between ACEs and bullying is mediated by depression and difficulty forming friendships. Moreover, a direct effect from ACEs to bullying was found. It was discussed that experiencing ACEs might make children more vulnerable to bullying. The relationship was moderated by gender [117]. This study provides further insight into the complex associations between bullying and ACEs, thereby providing additional support for the assumption drawn from the review of the selected studies that ACEs are a risk factor for bullying. This could be interpreted as lending support to the revictimization approach. Two of the three studies described previously found a dose-dependent relationship between the number of ACEs and victimization [70,72]. This phenomenon may be interpreted in one of two ways: as a case of revictimization, or as further evidence that bullying is an ACE, since the occurrence of one ACE has been demonstrated to elevate the risk of experiencing subsequent ACEs [92]. Another study revealed that bullying can moderate the protective effect of resilience. This study posits that individuals exposed to both ACEs and bullying exhibit elevated levels of psychopathology in comparison to those who have not been subjected to bullying, despite equivalent levels of resilience [118]. This finding, in addition to the aforementioned reviewed studies, underscores the significance of bullying as a contributing factor to mental health outcomes, in addition to the impact of ACEs. Moreover, connecting these results to the previous studies on extended ACEs, it was shown that bullying contributes to negative mental and physical health outcomes and predictions independently. Therefore, even if bullying is a result of experiencing other ACEs, including it in the framework would still lead to a more nuanced understanding of the emerging consequences. This is because it does add to the outcome in a manner similar to additional original ACEs. In accordance with this line of thinking, emotional neglect may also, in certain instances, be a consequence of parental substance use, parental mental illness, or parental separation. Nevertheless, it contributes to the dose-dependent relationship between exposure to ACEs and adverse health outcomes.

Among the studies employing LCA, all three studies identified a class characterized by a form of emotional neglect and abuse as well as bullying [73,74,75]. The findings indicate that bullying is associated with adverse health outcomes, particularly among children who have been subjected to emotional neglect or abuse in the domestic environment. Although LCA is an emerging method, not many studies could be found that include ACEs in conjunction with bullying to support or contradict the reviewed studies. However, a study conducted in Chile employed the ACE-IQ as a measurement. The ACE-IQ encompasses bullying. Consistent with the findings of preceding studies, the present investigation reveals that bullying is prevalent even within the low-adversity class, exhibiting a probability of approximately 20%. Moreover, bullying has been identified as a prevalent phenomenon across all four classes delineated in this study: low adversity, moderate adversity, high adversity primarily involving abuse, and polytrauma. The moderate adversity class was characterized by a low prevalence of abuse, yet a heightened occurrence of bullying and collective violence. No statistical differences regarding depressive symptoms could be found when comparing the three higher classes [119]. A subsequent study identified six classes, namely minimal ACEs, bullied emotionally, difficult homes, emotional neglect, parental and peer bullying and parental abuse and neglect. Contradictory to the findings of other studies, bullying was predominantly reported in the “bullied emotionally” and “parental and peer bullying” classes. Furthermore, it was determined that distinct classes are linked to disparate mental and physical health conditions. However, all classes exhibited a higher propensity to report health conditions in comparison to the minimal ACE class [120]. This finding may be interpreted as a minor inconsistency with the revictimization approach, supporting the conclusion drawn in the literature review, since bullying seems to contribute in a similar manner to long-term mental and physical health consequences. Nevertheless, bullying appears to be a salient factor in the development of children who have similar experiences in the domestic environment.

Research findings concerning biological stress markers have yielded mixed results. A study on BMI, inflammation, ACEs, and bullying revealed divergent results for ACEs and bullying, supporting the position that bullying might not be an ACE, because there seems to be differences in biological embodiment of the different experiences [77]. Further exploration into this matter is warranted, with the inclusion of research addressing the mechanism in future studies. At present, scientific knowledge regarding the mechanism is limited. Children who are overweight are at a higher risk of bullying [121,122]. Another study revealed enhanced BMI and higher inflammation levels for women subjected to bullying during their childhood [123]. A meta-analysis found a correlation between ACEs and increased childhood obesity levels; however, the impact of these factors may manifest over a period of years [124].

A synthesis of the studies included in this review suggests that the evidence from biological stress markers is inconclusive for both ACEs and bullying. However, it should be noted that other studies have yielded contradictory results.

In contrast to the findings of the aforementioned study by Marzi, there is evidence supporting the hypothesis that methylation is enhanced in both ACE and bullying research. Oulett-Morin compared bullied and non-bullied twins regarding cortisol response in the Trier Social Stress Test (TSST) and methylation at the SERT promoter region in a longitudinal study [125]. In this manner, the researchers were able to rule out the possibility that the observed variations among the twin pairs were preexistent. The investigation revealed that the twins subjected to bullying did not exhibit an elevated cortisol level in the TSST. However, a heightened level of methylation was observed. It was hypothesized that the hypothalamic–pituitary–adrenal (HPA) axis activity is disrupted because of an impaired serotonin neurotransmission. It is possible that this constitutes a mechanism that, in subsequent life, results in psychopathology associated with a modified cortisol response, including depression, externalizing problems, and PTSD. Comparable results were found for ACEs. Participants with ACE exposure exhibited decreased cortisol response in stressful situations and elevated levels of methylation [126,127,128]. It is important to acknowledge the slight disparities observed in the DNA regions. These variations may be indicative of alternative forms of embodiment or the influence of distinct methodologies.

Wankerl’s research yielded a lower SERT mRNA expression in adults who experienced childhood maltreatment, yet no methylation was detected in the corresponding DNA region [129]. This finding indicates that, at the molecular level, ACEs and bullying may manifest through analogous or potentially distinct pathways of embodiment, which could elucidate subsequent adverse health outcomes. Furthermore, there are several studies supporting Iobs et al.’s hypothesis that increased stress markers are detected for exposure period in late childhood rather than in response to a particular form of experience. Murphy’s research indicated an increase in CRP, IL-6 and suPAR at 24 years for childhood trauma experienced between ages 11–17, while early and middle childhood alone were not associated with a change in stress markers [130]. However, there was an association between the entire childhood period ACE expose [0–17] and both IL-6 and suPAR changes. Beijers’ observations revealed no alterations in children between the ages of six and ten [131].

Another marker of stress is telomere length. Telomeres can be found at the end of chromosomes to protect the DNA from erosion. A study has been conducted that established a correlation between social victimization and decreased telomere length, thereby emphasizing the impact of social forms of violence [132]. A similar finding was made in the context of ACE research. A meta-analysis of the extant literature revealed that six of eight studies found shorter telomeres after exposure to ACEs [133].

Further evidence has been obtained through brain image studies. A search of the extant literature yielded no studies that directly compared bullying and other ACEs. However, there are studies focusing on either bullying or ACEs. Childhood represents a vulnerable period in neuronal development, which may consequently provide a rationale for the subsequent emergence of mental health concerns. Connaughton et al. employed data from the International Longitudinal Imagen Project to investigate alterations in the brain subsequent to bullying experiences [134]. Alterations were identified for 49 brain regions, especially in areas in the frontal, parietal, occipital and temporal regions as well as the cerebellum. Reduction was especially noticeable in the left cerebral cortes, the bilateral insula and the right medial orbitofrontal cortex. An increase in volume was observed in limbic and subcortical regions, the left and right caudatus, the hippocampus, amygdala, accumbens and putamen. A discrepancy was detected in relation to gender. One potential explanation for this phenomenon is that females may engage more frequently in relational bullying, while males may engage more frequently in physical bullying. Comparable results were found in two earlier studies [135,136].

A comparable study was conducted on brain development and ACEs [137]. A series of alterations were identified in specific regions of the brain, including the anterior cingulate cortex, the dorsolateral prefrontal cortex, the orbitofrontal cortex, the corpus callosum, and the hippocampus. Furthermore, an elevated level of activity was observed in the amygdala region [137]. A comparison of the studies reveals that frequently, analogous regions are impacted by ACEs or bullying exposure, notably the orbitofrontal cortex, hippocampus, amygdala, and frontal cortex regions. The observation that mapping regions are affected lends credence to the hypothesis that analogous processing takes place in the brain. This finding suggests a potential parallel between ACEs and bullying.

Considering negative health outcomes, most of the reviewed studies did focus on mental health outcomes, while only three focused on physical health. An association has been identified for both ACEs and bullying regarding diabetes, pain levels and chronic diseases [52,79,80]. In addition to the aforementioned evidence on similarities regarding long-term physical health outcomes, a study revealed associations between worse oral health and bullying, sexual, physical and psychological abuse, parental death and parental divorce. The findings of the study indicated a positive correlation between the presence of any ACE and an elevated risk of suboptimal oral health outcomes [138]. It is important to note that the correlation between the distinct ACEs and health outcomes may vary, and not all ACEs are equally significant. A study conducted by Sweeting et al. aimed to investigate potential links between bullying, parental neglect, physical abuse, sexual abuse and witnessed parental violence and mental and physical health ailments in adulthood in a representative national sample. The results suggested that individuals who disclosed experiences of bullying and sexual abuse during their childhood were more likely to exhibit a higher prevalence of physical health ailments in comparison to those who did not have those adverse experiences. This offers further support for the conclusions drawn in the literature review and supports the hypothesis that bullying is an ACE. Moreover it was found that negative childhood experiences are linked to negative life events experienced in adulthood, which might contribute to the experienced distress and unfavorable mental health outcomes [139]. Bullying could be linked to a similar matter as ACEs in various mental health outcomes. This conclusion is supported by various studies in the field. Blanchflower and Bryson sought to examine the consequences of bullying, using cohort study data from a cohort born in 1958. The findings revealed a long-lasting impact on well-being, mortality and the likelihood of being unemployed in adulthood. This underscores the grave consequences of bullying, which can exert a profound influence on an individual’s future life trajectory [140]. Associations with mortality and lower socioeconomic status are also evident for ACEs [58,141,142]. This demonstrates a further similarity between the consequences of these two experiences, which could support the conceptualization of bullying as an ACE. In a subsequent publication, Blanchflower and Bryson utilized cross-sectional data spanning over two decades to map alterations in the mental well-being of young American citizens. They identified a general increase in mental health problems, along with strong associations between exposure to ACEs and poor mental health as well as a negative association for in-person bullying and cyberbullying and well-being [143]. Regarding negative mental health outcomes, an earlier onset of borderline personality disorder (BPD) was identified and positive correlations were found for ADHD and emotional problems for both ACEs and bullying [81,82,83,88].

In addition to the findings previously documented in this review, a curious finding was made regarding binge eating disorder in a sample of adults with obesity. A significant association between maltreatment and binge eating disorder was identified, while the association between bullying and binge eating was not significant. However, an indirect effect was identified, which was mediated by harm avoidance, self-directedness and maladaptive cognitive-emotional regulation. This association was posited as a potential explanation for the established link between bullying and binge eating. This finding is particularly interesting because it reemphasizes the potential for disparities in embodiment and underscores the necessity for further research to elucidate these effects. On the other hand, the study found that both the forms of maltreatment assessed in this study and bullying are associated with the suggested moderators, indicating a similarity between the experiences [144]. Bullying exhibited a comparable impact on the risk of self-harm and suicide as certain ACEs [84]. Corresponding findings were reported by Blair et al. and Liu et al. [145,146]. Blair et al. conducted a systematic review on the association between ACEs and suicidality in low-income countries. Although the original 10 ACEs were not employed, a slightly different set was utilized, which did include bullying. The results indicated that a multitude of adverse factors, including bullying, emotional, verbal, and psychological abuse, parental separation, divorce, or death, physical abuse or violence, physical attack, and sexual abuse, are associated with increased odds of suicidal outcomes [145]. Liu et al. conducted a study on non-suicidal self-injury. The study revealed a significant association between ACEs and self-injury, as well as a significant association between different forms of bullying and self-injury. Furthermore, research has revealed that bullying plays a partial mediating role in the association between ACEs and instances of self-injury. This finding indicates the presence of both a direct and an indirect effect [146]. The co-occurrence of bullying and ACEs and the comparable effect on self-harm lends further support to the proposition that bullying should be considered an ACE. However, a concurrent interpretation could be that bullying frequently constitutes a form of revictimization, stemming from earlier traumatic experiences in the victim’s childhood. Additionally, a recent study found a link between ACEs, including bullying and psychotic symptoms. Abuse, neglect and bullying were associated with positive psychotic symptoms, while general traumatic events, such as natural disaster or experiencing an accident were not associated with either negative or positive psychotic symptoms [147]. This finding lends further support to the hypothesis that bullying exhibits a greater degree of similarity to ACEs than to other traumatic events. It should be noted that several studies observed gender disparities [67,71,88]. Corresponding to that, a review was conducted on gender differences and ACEs in association with depression and anxiety. Results show that gender differences were found for original ACEs and for bullying as well. Women exhibit more anxiety compared to men when exposed to two ACEs, but less when exposed to emotional or sexual abuse or an incarcerated household member. Moreover, it was examined that men show higher odds of depression than women when exposed to bullying [148]. It seems that gender differences are a phenomenon not only limited to bullying, but ACEs in general.

## 4. Discussion

The aim of this review was to provide a comprehensive review of the extant literature on the current state of the art concerning the question of whether bullying should be considered an ACE. ACEs have been linked to a broad spectrum of adverse consequences. Therefore, it is challenging to respond definitively to the inquiry regarding the incorporation of bullying within the ACE framework using a solitary study. Therefore, the objective of this review was to synthesize the evidence across different categories, offer an overview of the milestones and the development of the understanding of ACEs and bullying, and demonstrate potential future directions. A comprehensive review of the extant studies reveals that bullying is associated with a variety of negative mental and physical health outcomes, as well as behavioral health outcomes comparable to those observed in original ACEs. Furthermore, it can be posited that ACEs exert divergent and deleterious effects when examined in isolation. However, when considered in the aggregate, ACEs have been demonstrated to predict a broad spectrum of outcomes. The salient point is that these factors cumulatively contribute to a negative burden, leading to adverse health consequences in the long and short term. Corresponding to previous associations established for the original ACEs, it is evident that comparable associations can be found for bullying, regarding both mental health outcomes like depression, borderline personality disorder, ADHD, suicidality, and physical health outcomes like pain, oral health, chronic diseases, and diabetes. The reviewed studies yielded mixed results regarding the relationship between bullying and behavioral health outcomes. However, other studies have indicated an association between bullying and behavioral health outcomes that resembles those of ACEs. Therefore, it can be concluded that there are similarities between ACEs and bullying in the outcomes frequently associated with ACEs. The collective evidence from the studies evaluated in this paper indicates that bullying plays a significant role in exacerbating this burden. Incorporating bullying into the ACE framework would facilitate the identification of at-risk children and adolescents, thereby enabling the delivery of more effective support measures. This conclusion is supported by several recent findings from various studies in the field. Despite a recent decline in reported instances of ACEs, including bullying, the challenges they pose to public health remain salient [149]. A recent study was conducted for the purpose of validating the Childhood Experience Survey. This measurement tool integrates the 10 original ACE and seven new ones, including bullying. All adversities exhibited a high degree of intercorrelation and were associated with symptoms of depression and anxiety, thereby underscoring their meaningful impact on mental health. It was concluded that an expanded ACE assessment might enhance the validity of ACE assessment and facilitate prevention and interventions [150]. Additionally, a study determined that the history of ACEs, as well as ongoing bullying, are significantly associated with increased rehospitalization rates across children and adolescents [151]. Corresponding to this, a study conducted in Finland found an association between bullying and both internalizing and externalizing disorders in an inpatient sample. In the inpatient group, bullying was identified as the second most prevalent ACE and was associated with all other ACEs. A correlation has been demonstrated between internalizing disorders and various ACEs, encompassing instances of physical abuse, parental substance use, parental mental health problems, and domestic violence. Similarly, an association has been observed between externalizing disorders and parental substance use, parental mental health problems, and physical abuse. The presence of both internalizing and externalizing disorders has been demonstrated to be associated with a higher number of ACEs [152]. This finding lends further support to the hypothesis that not all ACEs are equally correlated with adverse outcomes; rather, the cumulative score is the most significant predictor. Therefore, minor discrepancies between the embodiment of ACEs and bullying might not be sufficient to contradict the hypothesis that bullying is an ACE. The presence of synergies between bullying and ACEs extends beyond their impact on health outcomes. A recent study found that both bullying involvement and ACEs are correlated with school disengagement, defined as a lack of interest in studying and school activities. It was discussed that future studies should be conducted to determine whether bullying should be included as an ACE [153]. Another study was conducted on patterns of ACEs, bullying and social media use in school shooters. According to the findings of the study, 72% of the subjects experienced at least one instance of ACE, and 60% of the school shooters were subjected to bullying [154]. This finding underscores the importance of an exhaustive understanding of risk factors to identify at-risk students, offer interventions and protect them and others from negative outcomes.

This review underscores the extensive nature of the field of ACEs and bullying, highlighting the multitude of research areas that require consideration. In the interest of scientific rigor and replicability, it is essential that future studies make a concerted effort to replicate existing findings. This is of particular importance in cases of inconsistent outcomes, such as in the context of diabetes research or the analysis of biological stress markers. Moreover, the impact of both ACEs and bullying is best studied in longitudinal research. Frequently, studies are compelled to rely on retrospective data, which can be subject to confounding or bias. Recent advances in statistical methodology, such as logistic regression and community analysis, present novel approaches to variable control. These methodologies allow for the testing of different models and the identification of variables that exhibit collective tendencies. This could provide novel insights into the relationship between ACEs and bullying. It is recommended that these methods be utilized in future studies to further elucidate the association.

Furthermore, gender disparities were identified. Given their pervasiveness in both expanded and original ACEs, this domain warrants future research endeavors aimed at elucidating the underlying mechanisms and the divergent health outcomes, with the objective of enhancing prevention and treatment strategies.

The ACE construct should be proved for possible revision, since not only bullying is considered a possible ACE but also community violence or poverty as well as war or natural disaster. In the interest of future studies, it is recommended that individual ACEs be reported alongside cumulative scores, thereby facilitating a comparative analysis of adversities. Furthermore, it may be advantageous to calculate a cumulative score based on the original ACEs and a cumulative score based on both the original and expanded ACEs. This would facilitate a comparison of the two models and ensure an enhancement in prediction. This would enable the ACE concept to develop with social and cultural contexts, for example, the growing number of children with separated parents and more social acceptance for divorce as well as the tendency for more natural disasters in Europe, like floods. Furthermore, there has been a notable increase in the number of children who have experienced war in recent years. This phenomenon indicates that the presence of children who have been exposed to war in their early years is more prevalent in our society today than it was in previous years.

In order to facilitate a comprehensive evaluation and synthesis of disparate studies, it is imperative to establish a uniform understanding of the term “ACE.” It is important to note that other childhood experiences are often included in ACE measurement. However, original ACEs are excluded without an explanation, which results in sum scores that share a name but consist of different items. The utilization of an instrument such as the ACE-IQ would serve to formalize the research process, thereby enhancing its rigor and credibility.

### Limitations

The most noteworthy limitation is the approach chosen for the screening process, which may have introduced bias. In this review, the screening process was carried out by one author, and the selection of studies was approved by the second author. In optimal circumstances, a team of reviewers would be responsible for the screening and selection of articles. This approach would facilitate the collection of a range of opinions, which would then be compared during the decision-making process regarding the inclusion or exclusion of articles. The approach that was employed in this case was chosen in consideration of the constraints that were imposed by the available time and human resources. Additionally state-of-the-art reviews are shaped by the authors’ subjectivity.

A comprehensive search of three databases was conducted; nevertheless, it is plausible that pertinent studies were not identified due to the restriction of the search to these specific databases. However, the screening of the third database yielded only a limited number of new studies, which was the rationale for concluding the search at that stage. The quality of a review is contingent upon the quality of the underlying studies. It is conceivable that studies of high quality have never been published due to a publication bias, which may have resulted from the absence of findings that would corroborate the studies’ hypotheses.

## 5. Conclusions

In light of the aforementioned evidence consisting of similarities between ACEs and bullying across long- and short-term mental and physical health outcomes, it can be concluded that the extant literature reveals compelling evidence suggesting that bullying may indeed be considered an ACE within the scope of the knowledge available to us at this time. It is not feasible to eliminate the possibility that bullying may be considered a form of revictimization. Indeed, given the established role of power imbalance as a contributing factor in bullying incidents, it is plausible to hypothesize that children who have previously encountered ACEs may be particularly susceptible to victimization through bullying. However, it is crucial to acknowledge that this does not negate the harmful nature of bullying. Indeed, the extant research suggests that bullying contributes to the negative effect of cumulative ACEs and is often associated with negative health outcomes independently. In the context of daily clinical practice, it is imperative to identify risk factors associated with poor mental health outcomes, with the objective of implementing effective treatment strategies. Furthermore, the existence of a comprehensive database of established ACEs provides crucial insights to policymakers, informing their decision-making processes regarding the allocation of prevention resources. As posited by several authors, the ACE construct in total is the subject of a greater degree of attention than the single specific factors that it comprises. Bullying fulfills the criteria that is widely understood to describe an ACE: it as an experience in childhood that is linked to negative health outcomes and often described as trauma. The fundamental distinction between the two constructs is that original ACEs transpire within the domestic environment, whereas the consideration of harmful childhood experiences may necessitate an expansion of the scope beyond the familial context. This assertion is particularly salient in light of the extensive evidence demonstrating the deleterious consequences of bullying. It is strongly recommended that future studies, particularly longitudinal studies, include measurement of bullying alongside traditional ACEs to further examine the basis for considering bullying as an additional ACE.

## Figures and Tables

**Table 1:** Study quality rating (MMAT).

	Are the Participants Representative of the Target Population?	Are Measurements Appropriate Regarding both the Outcome and Intervention (or Exposure)?	Are There Complete Outcome Data?	Are the Confounders Accounted for in the Design and Analysis?	During the Study Period, is the Exposure Administered as Intended?
SmithBattle et al. (2022) [62]	review				
Karatekin and Hill (2019) [63]	no	yes	yes	yes	yes
Morrill et al. (2019) [64]	yes	yes	yes	yes	yes
Finkelhor et al. (2013) [65]	yes	yes	no	yes	yes
Sasaki et al. (2024) [66]	yes	yes	yes	yes	yes
Lampe et al. (2022) [67]	no	yes	yes	yes	yes
Folk et al. (2023) [68]	no	yes	no	yes	can’t tell
Smith et al. (2023) [69]	yes	yes	can’t tell	yes	yes
Merrin et al. (2024) [70]	review				
Sapouna et al. (2025) [71]	yes	yes	no	yes	can’t tell
Nagata et al. (2023) [72]	yes	yes	yes	yes	yes
Bond et al. (2023) [73]	no	yes	yes	can’t tell	yes
Liu et al. (2022) [74]	yes	yes	no	yes	yes
Hirai et al. (2025) [75]	yes	yes	yes	yes	yes
Marzi et al. (2018) [76]	yes	yes	no	yes	yes
Soares et al. (2022) [77]	yes	yes	can’t tell	yes	yes
Iob et al. (2022) [78]	yes	yes	yes	yes	yes
Gísladóttir et al. (2025) [79]	yes	yes	no	yes	yes
Brown et al. (2018) [52]	yes	yes	no	yes	yes
Njoroge et al. (2023) [80]	no	yes	no	yes	yes
Bozzatello et al. (2020) [81]	no	yes	yes	yes	yes
De Vries et al. (2024) [82]	yes	yes	can’t tell	yes	can’t tell
Schwartz et al. (2023) [83]	yes	yes	yes	yes	yes
Laporte et al. (2023) [84]	yes	yes	yes	yes	yes
Trompeter et al. (2024) [85]	yes	yes	no	yes	yes
Manoli et al. (2023) [86]	can’t tell	yes	yes	yes	yes
Wakuta et al. (2023) [87]	yes	yes	yes	yes	yes
Miller et al. (2024) [88]	can’t tell	yes	yes	yes	yes

**Table 2:** Overview of the results.

Category	Studies	Main Findings	Conclusions
Expanded ACEs	SmithBattle et al. (2022) [62]	Independent association between bullying and mental health outcomes, strongest predictor for health was the total ACE score to which expanded ACEs contribute	- inclusion of bullying into the ACE construct enhances predictive accuracy - additional influence of bullying on psychological distress - inconsistency regarding the independence of this effect from other ACEs
	Karatekin and Hill (2019) [63]	4-factor model, with the factors child maltreatment, household dysfunction, community dysfunction and peer dysfunction/property victimization accounting for 60% of total variance in health outcomes	
	Morrill et al. (2019) [64]	Including bullying in the model significantly improves the model fit. Bullying loaded on the factor “poor child environment”.	
	Finkelhor et al. (2013) [65]	Robust correlation between bullying and emotional distress. Dose-dependent relationship between ACEs and distress, whereby not all original ACEs contribute independently.	
	Sasaki et al. (2024) [66]	Significant impact of bullying on psychological distress. All ACEs except for parental death were associated with psychological distress and exhibit a dose-dependent relationship.	
	Lampe et al. (2022) [67]	The inclusion of bullying further enhances the predictive validity within the dose-dependent ACE–distress relationship. It is only independently associated with depression and somatization in the female sample but not in the male.	
	Folk et al. (2023) [68]	In youths with first-time court involvement, a high prevalence of ACEs and bullying was found. Expanded ACEs were not associated with behavioral health outcomes, but trauma and externalizing and internalizing symptoms.	
	Smith et al. (2023) [69]	Bullying was prevalent across all classes formed in the LCA, and the classes with more ACEs exhibited an enhanced risk for cannabis use. A significant correlation between CUD and bullying was revealed.	
Associations between ACEs and bullying	Merrin et al. (2024) [70]	Regarding bullying victimization, positive correlations were identified for cumulative ACEs, physical abuse, emotional abuse, household violence, maltreatment, family violence, and neglect	- exposure to ACE is a risk factor for bullying victimization - could support the revictimization theory - or support the assumption that bullying is an ACE, since experiencing one ACE is a risk factor for other ACEs
	Sapouna et al. (2025) [71]	In a longitudinal study conducted in Scotland, bullying was almost as prevalent as the most common ACE. The presence of one ACE enhanced the risk of becoming a bullying victim.	
	Nagata et al. (2023) [72]	A dose–response relationship was found between the number of ACEs and cyberbullying victimization. Associations between single ACEs and victimization were identified.	
Latent class analysis	Bond et al. (2023) [73]	A bullying class was identified alongside a high and a low adversity class. Bullying was prevalent across all classes, and the bullying class showed the highest levels of depression and suicidal ideation. Bullying improved the explanatory power of the model.	- all studies identified a high-, a low-adversity class and a bullying and emotional abuse or neglect class - bullying as an important addition to the original ACEs - prevalence across all classes contradicts the revictimization theory
	Liu et al. (2022) [74]	An emotionally neglected and bullied class was identified. Social support is able to buffer the impact of ACEs.	
	Hirai et al. (2025) [75]	A high ACE, a low ACE, a poverty and a psychological abuse class, which included bullying, were identified. The high ACE class had the strongest association with adverse physical and mental health effects and abuse-related behavior, but it was shown that even individuals in the low adversity class experience negative effects.	
Biological stress markers	Marzi et al. (2018) [76]	No associations between DNA methylation and any form of victimization were found.	- only limited support for the hypothesis - alternative modes of embodiment could imply that bullying may not be an additional ACE
	Soares et al. (2022) [77]	A predominantly direct impact on hs CRP was identified for ACEs and a predominantly indirect impact for BMI in cases of bullying. This suggests different paths of embodiment.	
	Iob et al. (2022) [78]	Factor analysis resulted in a physical/emotional threat group and a household dysfunction group of ACEs, while sexual abuse and bullying remained as standalone factors. The only exclusive associations for CPR were found for bullying and sexual abuse in boys. Higher CRP levels were evident in the household dysfunction group among adolescents.	
Negative health outcomes associated with ACEs and bullying	Gísladóttir et al. (2025) [79]	ACE exposure predicts increased risk of diabetes type 2 by approximately 10% for each additional ACE. In a model controlled for adult BMI and other ACEs, bullying emerged as the strongest predictor, followed by sexual abuse and mental illness of a household member.	- positive correlation between bullying and negative health outcomes - resemble those observed for original ACEs
	Brown et al. (2018) [52]	Individual ACEs demonstrate varying degrees of correlation with disparate outcomes of pain measurements. Bullying showed a moderate correlation with any pain, a weak correlation comparable to physical neglect with bodily pain and the third strongest correlation with headache.	
	Njoroge et al. (2023) [80]	Bullying shows comparable results to several original ACEs regarding chronic diseases. An association between bullying and hypertension, hyperlipidemia and dysglycemia was revealed as well as having one or three chronic diseases.	
	Bozzatello et al. (2020) [81]	A correlation between the earlier onset of borderline personality disorder and adverse childhood experiences, such as abuse, neglect, household dysfunction, and bullying, was found.	
	De Vries et al. (2024) [82]	Bullying was identified as the sole ACE that was grouped with emotional problems and remained a relevant factor when controlled for exposure to other ACEs. Another result was that the original ACEs are not a homogeneous construct; rather, they exhibit differential impacts.	
	Schwartz et al. (2023) [83]	It was found that each ACE elevates the risk for ADHD. The strongest associations were identified for sexual abuse, both forms of neglect and bullying.	
	Laporte et al. (2023) [84]	It was found that the presence of each additional ACE, including bullying, enhanced the risk of self-harm.	
	Trompeter et al. (2024) [85]	Both bullying and ACEs independently contribute to internalizing and externalizing symptoms and a dose-dependent relationship was demonstrated.	
	Manoli et al. (2023) [86]	The association between bullying and worse clinical expressions of bipolar disorder persists even if maltreatment as a covariate is included. There appears to be an additive relationship between bullying and other forms of maltreatment regarding suicidal behavior.	
	Wakuta et al. (2023) [87]	The prevalence and enhancement of depression risk was found to be higher for school ACEs than for original ACEs. Bullying was the strongest predictor for hikikomori.	
	Miller et al. (2024) [88]	A total of 28% of the participants identified bullying as the most aversive experience. The path analysis on emotional distress was significant for both bullying and cyberbullying in females and only for cyberbullying in males. For both genders, an interaction of bullying and ACEs was found.	

## Data Availability

No new data was created for this study.

## Abbreviations

The following abbreviations are used in this manuscript:

ACE	Adverse Childhood Experience
ADHD	Attention deficit hyperactivity disorder
BMI	Body mass index
BPD	Borderline personality disorder
CFA	Confirmatory factor analysis
CUD	Cannabis use disorder
DNA	Deoxyribonucleic acid
EFA	Exploratory factor analysis
HPA	Hypothalamic–Pituitary–Adrenal Axis
Hs CRP	High-sensitivity C-reactive protein
JVQ	Juvenile Victimization Questionnaire
LCA	Latent class analysis
PTSD	Post-traumatic stress disorder
TSCC	Trauma Symptoms Checklist for Children
TSST	Trier social stress test
WHO	World Health Organization
